# Observational study to assess pregnant women’s knowledge and behaviour to prevent toxoplasmosis, listeriosis and cytomegalovirus

**DOI:** 10.1186/1471-2393-13-98

**Published:** 2013-04-30

**Authors:** Monique T R Pereboom, Judith Manniën, Evelien R Spelten, François G Schellevis, Eileen K Hutton

**Affiliations:** 1 Department of Midwifery Science, AVAG and the EMGO+ Institute for Health and Care Research, VU University Medical Center, P.O. Box 7057, (Room D4.40), 1007 MB Amsterdam, The Netherlands; 2 Netherlands Institute for Health Services Research (NIVEL), P.O. Box 1568, 3500 BN Utrecht, The Netherlands; 3 Department of General Practice and Elderly Care Medicine/EMGO+ Institute for Health and Care Research, VU University Medical Center, P.O. Box 7057, (Room D5.38), 1007 MB Amsterdam, The Netherlands; 4 Faculty of Health Sciences, McMaster University, Michael G. DeGroote Centre for Learning (Room 2210), 1280 Main St. W. Hamilton, Ontario L8S 4K1, Canada

**Keywords:** Infectious diseases, Toxoplasmosis, Listeriosis, Cytomegalovirus, Prenatal health care providers, Pregnant women, Knowledge, Risk behaviour, Prevention

## Abstract

**Background:**

Toxoplasmosis, listeriosis and cytomegalovirus (CMV) can negatively affect pregnancy outcomes, but can be prevented by simple precautions of pregnant women. Literature suggests that pregnant women are not always adequately informed by their care provider about preventable infectious diseases and most pregnant women have a low level of knowledge regarding these topics. There is not much information about the actual risk behaviour of pregnant women. The purpose of this study was to assess knowledge and risk behaviour related to toxoplasmosis, listeriosis and CMV infection prevention in pregnant women.

**Methods:**

A cross-sectional survey among pregnant women from twenty midwifery practices across the Netherlands that participated in the DELIVER study, between October 2010 and December 2010. The questionnaire items covered respondents’ knowledge of preventive practices in general, risk behaviour, and sources of received information.

**Results:**

Of the 1,097 respondents (response 66.0%), 75.3% had heard, read or seen information about toxoplasmosis, 61.7% about listeriosis and 12.5% about CMV. The majority reported having heard about these infections from their care providers or read about these in printed media or on the Internet. Respondents showed limited knowledge about preventive practices for toxoplasmosis, listeriosis or CMV infection. Regarding toxoplasmosis, risk behaviour was more prevalent among respondents who had a high level of education, had the Dutch nationality, did not take folic acid during their first trimester, and had ever worked in a children day-care setting. Regarding listeriosis, risk behaviour was more prevalent among respondents who where in their third trimester. Regarding CMV infections, risk behaviour was less prevalent among respondents who were in their third trimester of pregnancy.

**Conclusion:**

Of the respondents, a substantial part did not have knowledge about preventive practices to avoid listeriosis, toxoplasmosis and CMV infections during pregnancy. Many pregnant women are appropriately avoiding risk behaviour, without knowing what they are avoiding. Advising pregnant women about behaviours and life-style habits to prevent infectious diseases remains important and information about preventive practices need to be complete and adequate. However, it may be less important to give pregnant women specific infectious diseases information. More attention towards CMV is necessary.

## Background

Pregnancy complications associated with *Toxoplasma gondii*, Listeria *monocytogenes* and cytomegalovirus (CMV) infections are prevented by simple precautions and behaviours of pregnant women [1-3] (Table 1). Many pregnant women are not aware of the risk and consequences of infectious diseases and are not practicing preventive strategies [3-9]. Studies from the United States have shown that most women of childbearing age and pregnant women had a limited knowledge of methods to prevent toxoplasmosis, listeriosis and CMV infections [3,5-7]. Studies about listeriosis revealed that women who had never heard of listeriosis were more likely to be young, be a single mother, live in rural areas, speak a foreign language at home, or have less formal education. In addition, knowledge levels of listeriosis among pregnant women differ between ethnic groups [5,10].

**Table 1:** Toxoplasmosis, listeriosis and CMV infections

	
Toxoplasmosis	Toxoplasmosis is caused by the protozoa *Toxoplasma gondii*[11] which can be transmitted by the pregnant woman to the foetus. Infection can occur through ingestion of viable tissue cysts in undercooked meat or through contact with oöcysts excreted by cats in the environment [12]. The incidence rate of congenital toxoplasmosis in the Netherlands is two infected children per 1000 live births. This is ten times higher than in Denmark [13], for example, and twenty times higher than in Ireland [14]. Primary infection with *Toxoplasma gondii* in pregnancy can lead to severe illness in the foetus and infant, including chorioretinitis, deafness, microcephaly, developmental delay and even stillbirth [15,16]. Transmission is rare in early pregnancy and increases with duration of pregnancy. Transmission frequency is approximately 15% in the first trimester, 30% in the second trimester and 60% in the third trimester of pregnancy [15]. Exposure to infection acquired in the first trimester causes more severe congenital illness and foetuses exposed in the third trimester are more likely to be asymptomatic at birth [15].
Listeriosis	Listeriosis, a food-borne infection caused by the bacteria *Listeria monocytogenes* is 17 times more likely to occur in pregnant women than in the general population [11,17]. Outbreaks of listeriosis occur mainly by consuming unpasteurized dairy products, smoked fish and ready-to-eat foods [17]. In the Netherlands, the estimated incidence rate of pregnancy-related listeriosis is between 1.3 and 2.4 cases per 100,000 pregnancies over 24 weeks of gestation [18,19]. Even though listeriosis is a rare disease, it can have serious consequences. Twenty percent of pregnancies complicated by listeriosis end in spontaneous abortion or stillbirth, and two-thirds of surviving infants develop clinical neonatal listeriosis. Moreover, listeriosis has a high case-fatality rate: 20–30 neonatal deaths per 100 cases of illness [11,17].
Cytomegalovirus	A cytomegalovirus (CMV) infection is the most common viral infection during pregnancy and the most common cause of congenital defects in newborns [11], with an estimated worldwide prevalence of 6.4 cases per 1,000 births [20]. Transmission occurs through contact with infected body fluids [21]. The incidence rate of congenital CMV in pregnant women in the Netherlands is 5.4 cases per 1000 [22]. In 30 to 40% of the pregnant women with a primary infection and in less than one percent of recurrent infections the foetus will be infected [23]. Ten to 15 percents of the infected foetuses have symptoms of the disease at birth (e.g. hepatosplenomegaly, intracranial calcification, chorioretinitis). Another 15–20 percent of the infected foetuses will develop symptoms during their first years of life (e.g. physical and mental retardation or hearing loss) [23]. A common way to require a CMV infection is through close contact with young children, who can secrete the virus in their saliva and urine for many months after their first infection. Women who are working in a child day care setting and women who have young children run a higher risk of infection [3].

There is not much information about the frequency with which pregnant women practice preventive behaviours for toxoplasmosis, listeriosis and CMV infections. While knowledge is an important determinant to establish behavioural change, accurate knowledge may not lead to appropriate preventive behaviour. Attitudes of pregnant women towards changing their behaviour, and their perception about the likelihood of contracting the infectious disease during their pregnancy may also be important contributors to establish behavioural change [24,25].

The objective of this survey was to assess knowledge of and risk behaviour related to toxoplasmosis, listeriosis and CMV infection in pregnant women, who are cared for by a primary care midwife in the Netherlands, and to determine which demographical characteristics are related to knowledge and risk behaviour. In addition, to determine differences in risk behaviours within sources of received information. We hypothesize that more knowledge regarding infectious disease prevention is associated with less risk behaviour in pregnant women.

## Methods

To assess pregnant women’s knowledge and behaviour towards infectious diseases we developed a self-administered questionnaire for this observational survey. Survey questions were divided per infectious disease (toxoplasmosis, listeriosis and CMV infections). In total, 50 questions covered respondents’ knowledge of preventive practices in general, risk behaviour, and sources of received information. Questions were based on previous studies [3-8,17,26-28] and the questionnaire took approximately 20 minutes to complete. The first section of the questionnaire covered knowledge about preventive practices. Respondents were presented with twelve (eight true and four false) preventive practices about the three diseases and were asked to tick an applicable box, including a ‘yes’, ‘no’ and ‘don’t know’ option. Each correctly identified preventive practice contributed to the knowledge score (one point for each correct answer). The knowledge score could therefore vary between zero and six for toxoplasmosis, between zero and three for listeriosis and between zero and three for CMV. The second section focused on the actual behaviour of pregnant women to prevent specific infectious diseases during their pregnancy. Only respondents who had children aged less than five years answered the questions about risk behaviour regarding CMV infections.

This survey was embedded in the DELIVER study, which is a large scale national survey into primary care midwifery in the Netherlands [29]. The DELIVER study took place in twenty primary midwifery care practices across the country and clients were recruited from these practices. Purposive sampling was used to select the midwifery practices, using three stratification criteria: region (north, east, south, west), level of urbanisation (urban versus rural area), and practice type (dual or group practice. Data collection of the DELIVER study took place between August 2009 and March 2011. The study protocol of the DELIVER study was approved by the Medical Ethics Committee of the VU University Medical Centre Amsterdam. Client participation was voluntary and they could withdraw at any time. Privacy was guaranteed in accordance with Dutch legislation. Clients’ anonymity was maintained by using anonymous participant and practice identifiers.

Demographic and pregnancy related information including the respondents’ age, number of pregnancies experienced, nationality, level of education, language spoken at home, living in a deprived area, planned or unplanned pregnancy, single/marital status, folic acid use and smoking behaviour was obtained via linkage to the questionnaires of the DELIVER study by an anonymized participant identifier. Nationality was determined by the respondents’ reported nationality and was dichotomized (Dutch nationality versus non Dutch nationality), because of small numbers of non-Dutch participants. Highest achieved educational level was determined and defined in three groups for analysis: low level of education (medium- level secondary education or below), medium level of education (higher-level secondary education or vocational education) and high level of education (diploma level or university education). The gestational age of the respondent was dichotomized into first or second trimester versus third trimester, because of small numbers of respondents who were in their first trimester. Finally, the number of pregnancies a respondent experienced was categorized into first (primigravidae) and multiple pregnancies (multigravidae).

From October to December 2010 we asked all participants in the DELIVER study, who were still pregnant at the time of survey completion, to fill in the additional questionnaire about toxoplasmosis, listeriosis and CMV infections. The questionnaire was sent to the home addresses of pregnant women with a return envelope. One reminder was sent to all non-responders after one month. Respondents were excluded from this survey if the link with the questionnaire of the DELIVER study that included demographic data could not be made.

The main outcome measures of this survey were knowledge about preventive practices and risk behaviour regarding toxoplasmosis, listeriosis and CMV infections of pregnant women. Knowledge and risk behaviour were assessed for the three infectious diseases separately. Frequency distributions for the questionnaire items on knowledge of preventive practices and risk behaviour were calculated. We used non-parametric tests, namely the Mann–Whitney *U* test and Kruskal Wallis test, to investigate differences in median knowledge scores per infectious disease between respondents’ characteristics. Non-parametric tests were used because the hypothesis of normally distributed data was rejected according to the Kolmogorov-Smirnov test at a 5% significance level. To detect any differences in respondents risk behaviour per preventive practice and within the received information (health care professional versus other source of information) we used Chi square tests for independence. To detect any associations between demographic variables and risk behaviour, we considered risk behaviour to be present if a respondent reported she had undertaken at least one of the behaviours during her current pregnancy which could increase the risk for one of the three infectious diseases. To investigate the association between demographic variables and risk behaviour of respondents, univariate logistic regression was used. Missing data were less than two percent for all demographic variables. Variables with a p-value of 0.10 in univariate analyses or less were included in a multivariate logistic regression model to control for potential confounding, using a manual backward selection procedure. Due to the hierarchical structure of the data (respondents clustered in midwifery practices), all analyses were repeated in multilevel analyses which revealed no significant cluster effects. The variables in the final model were presented as odds-ratios (OR) with 95% confidence intervals (CI). A two-tailed p-value of 0.05 or lower was considered statistically significant. The statistical software package SPSS 18.0 (SPSS inc., Chicago, IL) was used for all data-analyses.

## Results

A total of 1,663 respondents who participated in the DELIVER study were invited between October 2010 and December 2010 to complete the questionnaire on infectious diseases. In total 1,123 (67.5%) respondents completed the questionnaire. Of these, we excluded 26 questionnaires which could not be linked to the demographic data. Data of 1,097 respondents were included in the analyses, representing a net response rate of 66.0%.

Respondents were more likely to have the Dutch nationality (96.9%) than the general Dutch population (93%), and were more likely to have a high education (56.9%) than the general Dutch population (17.1%) [30,31]. Other demographic characteristics of the study population are presented in Table 2.

**Table 2:** Characteristics of pregnant women and median knowledge score per infectious disease; N = 1,097

	**Total**	**Toxoplasmosis**		**Listeriosis**		**CMV**	
**Determinants**	N^* ^**(%)**	**Median knowledge score**^**2**^	**p-value**^**3**^	**Median knowledge score**^**2**^	**p-value**^**3**^	**Median knowledge score**^**2**^	**p-value**^**3**^
Age							
17-25 year	148 (13.5)	4.0	0.27	1.0	.000*	0.0	.40
26-34 year	761 (69.4)	4.0		2.0		0.0	
35-42 year	188 (17.1)	4.0		2.0		0.0	
Education^1^							
Low	116 (10.6)	3.0	.000*	1.0	.000*	0.0	.53
Medium	356 (32.5)	4.0		1.0		0.0	
High	624 (56.9)	4.0		2.0		0.0	
Other nationality	34 (3.1)	3.0	.72	2.0	.24	0.0	.004*
Dutch nationality	1061 (96.9)	4.0		2.0		0.0	
Dutch spoken at home	1073 (97.9)	4.0	.99	2.0	.60	0.0	.05*
Other language than Dutch spoken at home	23 (2.1)	3.0		2.0		0.0	
Not living in a deprived area	1062 (96.8)	4.0	.64	2.0	.48	0.0	.09
Living in a deprived area	25 (3.2)	3.0		1.5		0.0	
Planned pregnancy	931 (85.3)	4.0	.00**	2.0	.005*	0.0	.78
Unplanned pregnancy	161 (14.7)	3.0		1.0		0.0	
Partner/married	1084 (98.9)	4.0	.001*	2.0	.008*	0.0	.29
Single	12 (1.1)	0.0		0.0		0.0	
1^st^ en 2^nd^ phase of pregnancy	462 (42.1)	4.0	.19	2.0	.16	0.0	.74
3^th^ phase of pregnancy	635 (57.9)	4.0		2.0		0.0	
Primigravidae	409 (37.8)	4.0	.95	2.0	.044*	0.0	.53
Multigravidae	672 (62.2)	4.0		2.0		0.0	
Did take folic acid in 1^st^ trimester	1039 (95.1)	4.0	.09	2.0	.22	0.0	.25
Did not take folic acid in 1^st^ trimester	53 (4.9)	3.0		1.0		0.0	
Did not smoke during pregnancy	1026 (94.0)	4.0	.002*	2.0	.000*	0.0	.53
Smoked during pregnancy	66 (6.0)	3.0		1.0		0.0	
Never worked as a health care professional	710 (64.8)	3.0	.000*	1.0	.000*	0.0	.000*
Ever worked as a health care professional	386 (35.2)	4.0		2.0		0.0	
Never worked as a child day care worker	959 (87.5)	4.0	.35	2.0	.71	0.0	.59
Ever worked as a child day care worker	137 (12.5)	3.5		1.5		0.0	

* Denominator varies due to missing values (between 0 and 10 missings per variable).

^1^ Low level of education: medium- level secondary education or below; medium level of education: higher level secondary education or vocational education; high level of education: diploma level or university education.

^2^ Maximum possible scores: toxoplasmosis = 6; listeriosis = 3; CMV = 3.

^3^ Differences in knowledge scores per characteristic (Mann–Whitney *U* test and Kruskal-Wallis test).

### Preventive practice knowledge

Of the 1,097 respondents, 75.3% (n = 794) had heard, read or seen some information about toxoplasmosis, 61.7% (n = 649) about listeriosis and 12.5% (n = 131) about CMV. Of these, the majority reported having heard about the infections from their health care provider or read about these in magazines, books, newspapers or on the Internet (Table 3).

**Table 3:** Sources where pregnant women get their information about infectious diseases from; N = 1.097

**Source**	**Toxoplasmosis (%)**	**Listeria (%)**	**CMV (%)**
**Health care professional**	48.3	38.7	3.4
**Internet**	41.6	35.5	5.9
**Newspaper, book or magazine**	37.3	30.2	2.6
**Family or friends**	17.0	10.7	1.4
**Other source**	4.3	3.1	2.5
**Radio or television**	3.2	2.7	0.4
**I did not see, hear or read anything about this disease**	24.7	38.3	87.5

Percentages do not sum to 100%, because multiple answers were accepted.

Knowledge about how to prevent toxoplasmosis and listeriosis varied by topic (Table 4). In terms of preventing toxoplasmosis, respondents were most likely to correctly indicate that avoiding changing the cat’s litter box (77.9%) and gardening with gloves (74.3%) were methods to prevent toxoplasmosis in pregnancy. Almost half of the respondents knew that not eating rare or undercooked meat (48.1%) and thoroughly washing and peeling fruits and vegetables (48.2%) are ways to prevent toxoplasmosis.

**Table 4:** Pregnant women’s knowledge about preventive practices for infectious diseases; N = 1.097

**Preventive practices**		**Correct answer (%)**	**False answer (%)**	**Don’t know (%)**
**Toxoplasmosis**	Letting someone else change the cat’s litter box	77.9	0.6	21.6
	Cooking meat well until no pink is seen and the juices run clear	48.1	12.7	39.3
	Thoroughly washing and/or peeling fruits and vegetables before eating them	48.2	13.3	38.5
	Gardening with gloves	74.3	1.3	24.4
	Removing pet reptiles from your home*	20.1	5.9	73.9
	Taking a vitamin supplement regularly*	47.6	3.3	49.2
**Listeriosis**	Thoroughly reheating all leftover foods and “ready-to eat” foods	45.8	9.9	44.3
	Eating only properly pasteurized dairy products	63.9	4.5	31.6
	Avoiding areas where ticks live*	32.2	2.2	65.6
**CMV**	Hand washing after diaper change	16.0	2.8	81.2
	Not sharing the same drinking glass, utensils or toothbrush with children	15.0	1.6	83.4
	Do not lick envelopes*	9.8	1.6	88.6

* False statement.

Of the respondents, 63.9% (n = 691) correctly identified that listeriosis can be prevented by avoiding the consumption of unpasteurized dairy products. Fewer respondents (45.8%) were aware that listeriosis can be prevented by thoroughly reheating all leftover foods and ready-to-eat foods.

Some statements were false (on purpose) and respondents seemed unsure about the answers to false statements for both toxoplasmosis and listeriosis prevention practices. For example, 73.9% (n = 797) of the respondents were unsure whether removing pet reptiles from your home would prevent toxoplasmosis and 65.6% (n = 707) of the respondents were unsure whether listeriosis could be prevented by avoiding areas where ticks live.

Among the three infectious diseases, knowledge about CMV ranked lowest, with the fewest correct answers and many respondents answering ‘don’t know’ for the statements related to CMV. Of the three statements regarding CMV prevention practices, the statement about hand washing after diaper change was most often answered correctly (16%).

Regarding toxoplasmosis, 9.4% (n = 103) of the respondents answered all six statements correctly and 20.0% (n = 218) answered none of the statements correctly; the median knowledge score was 4.0 (5^th^ percentile 0.0, 95^th^ percentile 6.0). Regarding listeriosis, 17.2% (n = 187) of the respondents answered all three statements correctly and 27.9% (n = 303) answered none of the statements correctly; the median knowledge score was 2.0 (5^th^ percentile 0.0, 95^th^ percentile 3.0). Regarding CMV, 5.0% (n = 54) of the respondents answered all three statements correctly and 79.2% (n = 863) answered none of the statements correctly; the median knowledge score was 0.0 (5^th^ percentile 0.0, 95^th^ percentile 2.5).

Table 2 shows the median knowledge scores per characteristic of the respondents. A higher median knowledge score for preventive practices for toxoplasmosis was associated with a higher level of education, a planned pregnancy, having a partner, not smoking during pregnancy and ever having worked as a health care professional. Regarding preventive practices for listeriosis, a higher knowledge score was associated with being 26 years or older, a higher level of education, a planned pregnancy, having a partner, multigravidity, not smoking during pregnancy and ever having worked as a health care professional. A higher median knowledge scores for preventive practices for CMV was associated with a non-Dutch nationality, speaking another language than Dutch at home, and ever having worked as a health care professional.

### Risk behaviour

We compared respondents’ risk behaviour during their current pregnancy with their knowledge about each corresponding preventive practice (Table 5). In general, the reported risk behaviour by respondents was most often associated with their knowledge of preventive practices of toxoplasmosis and listeriosis. Even though only 48.1% (n = 520) of the respondents knew that eating rare or medium cooked meat increases the risk for a toxoplasmosis infection, most of them did not eat this during pregnancy (91.7%).

**Table 5:** Risk behaviour*; Overall and separately for women with correct and incorrect/no knowledge of preventive practices

**Risk behaviour**		**Overall**	**Incorrect/No knowledge**	**Knowledge**
		**(%)**	**(%)**	**(%)**
**Toxoplasmosis N = 1.097**	Eat rare or medium cooked meat	8.3	7.7	8.7
	Garden without gloves	21.4	20.9	21.8
	Change the cat litter box	4.4	1.2	5.3
	Eat unwashed raw vegetables or fruits	46.7	50.6	42.2
**Listeriosis N = 1.097**	Eat unpasteurized dairy products	8.2	9.5	7.5
	Eat ready to eat foods	40.4	39.5	41.9
**CMV N = 541**	Share utensils or cups with children	91.3	92.0	87.8
	Did not wash hands after diaper change	69.4	70.8	61.5

* Risk behaviour is defined as having done it at least once during their current pregnancy.

There were almost no differences in reported risk behaviour between respondents who knew that eating raw or medium cooked meat, eating unwashed fruits and vegetables and gardening without gloves was a preventive practice for toxoplasmosis and respondents who had incorrect or no knowledge about these preventive practices. With regard to listeriosis, only 8.2% (n = 90) reported ever having consumed an unpasteurized dairy product. These percentages are comparable with the knowledge level respondents had about these topics. Few respondents reported adapting their behaviour to prevent a CMV infection. Of all 541 respondents who had children less than five years of age in their household, 91.3% (n = 480) reported to have shared utensils or cups with their children at least once during their pregnancy and 69.4% (n = 366) did not wash their hands at least once after changing a diaper. This was also comparable with the level of knowledge respondents had about CMV (Table 5). We did not find a difference in the percentage of respondents who undertook a certain risk behaviour between respondents who received information on the infectious disease from their health care professional versus other sources of information.

We examined whether demographic characteristics were correlated with risk behaviour towards toxoplasmosis, listeriosis and CMV infections during pregnancy. Of the 1,097 respondents, 59.1% (n = 642) reported at least one risk behaviour for toxoplasmosis and 46.0% (n = 501) reported at least one risk behaviour for listeriosis. Of the 541 respondents who had children aged less than five years living in their household, 95.5% (n = 504) reported at least one risk behaviour for CMV during their current pregnancy. The demographic variables ‘Other language than Dutch spoken at home’, ‘Single’ and ‘Did not take folic acid’ were excluded from the logistic regression analysis regarding CMV infection due to small numbers. Detailed information on background characteristics of respondents who had children less than five years living in their household are shown in Table 6.

**Table 6:** **Associations between characteristics of pregnant women and their risk behaviour**^**1 **^**regarding toxoplasmosis, listeriosis or CMV**

	**Toxoplasmosis**			**Listeriosis**			**CMV**			
**Determinants**	**Risk behaviour (%)**	**OR (95% CI)**	**Adjusted OR (95% CI)**	**Risk behaviour (%)**	**OR (95% CI)**	**Adjusted OR (95% CI)**	**N = 541 (%)**	**Risk behaviour (%)**	**OR (95% CI)**	**Adjusted OR (95% CI)**
Age										
17-25 year	51.4	-		28.8	-		6.8	97.3	-	
26-34 year	59.9	1.4 (1.0-2.0)*		46.9	1.4 (1.0-2.0)*		72.1	96.6	0.8 (0.1-6.2)	
35-42 year	62.0	1.6 (1.0-2.4)*		47.8	1.5 (0.9-2.3)		21.1	91.1	0.3 (0.0-2.3)	
Education^2^										
Low	52.2	-	-	41.4	-		10.0	94.2	-	
Medium	52.8	1.0 (0.7-1.6)	1.1 (0.7-1.6)	42.5	1.1 (0.7-1.6)		32.0	94.6	1.1 (0.3-4.2)	
High	63.9	1.6 (1.1-2.4)*	1.7 (1.1-2.6)**	48.9	1.4 (0.9-2.0)		58.0	96.1	1.5 (0.4-5.6)	
Non Dutch nationality	43.8	-	-	41.2	-		2.8	93.3	-	
Dutch nationality	59.6	1.9 (0.9-3.9)*	2.1 (1.0-4.3)**	46.2	1.2 (0.6-2.5)		97.2	95.5	1.5 (0.2-12.7)	
Dutch spoken at home	59.1	-		46.2	-		98.7	95.4	-	
Other language than Dutch spoken at home	57.1	0.9 (0.4-2.2)		39.1	0.8 (0.3-1.7)		1.3	100.0	***	
Not living in a deprived area	59.4	-		45.7	-		98.5	95.6	-	
Living in a deprived area	51.4	1.4 (0.7-2.7)		54.3	1.4 (0.7-2.8)		1.5	87.5	0.3 (0.0-2.7)	
Planned pregnancy	59.3	-		46.5	-		85.3	95.1	-	
Unplanned pregnancy	57.6	0.9 (0.7-1.3)		42.1	0.8 (0.6-1.2)		14.7	97.4	2.0 (0.5-8.9)	
Partner/married	59.2	-		46.0	-		99.3	95.4	-	
Single	50.0	0.7 (0.2-2.2)		50.0	1.2 (0.4-3.7)		0.7	100.0	***	
1^st^ en 2^nd^ phase of pregnancy	60.5	-		41.4	-	-	41.2	97.7	-	-
3^th^ phase of pregnancy	58.1	0.9 (0.7-1.2)		49.3	1.4 (1.1-1.8)*	1.4 (1.1 – 1.8)**	58.8	93.9	0.4 (0.1-1.0)*	0.4 (0.1 – 1.0)**
Primigravidae	56.5	-		48.5	-		0.4	100.0	-	
Multigravidae	60.6	1.2 (0.9-1.5)		44.1	0.8 (0.7-1.1)		99.6	95.4	***	
Did take folic acid in 1^st^ trimester	58.2	-	-	45.8	-		94.0	95.1	-	
Did not take folic acid in 1^st^ trimester	76.0	2.3 (1.2-4.4)*	2.7 (1.4-5.3)**	47.2	1.1 (0.6-1.8)		6.0	100.0	***	
Did not smoke during pregnancy	58.6	-		46.1	-		95.3	95.4	-	
Smoked during pregnancy	65.2	1.3 (0.8-2.2)		43.1	0.9 (0.5-1.5)		4.7	96.0	1.2 (0.2-9.0)	
Never worked as a health care professional	59.3	-		46.5	-		64.1	95.6	-	
Ever worked as a health care professional	58.9	1.0 (0.8-1.3)		45.1	0.9 (0.7-1.2)		35.9	95.2	0.9 (0.4-2.2)	
Never worked as a child day care worker	57.8	-	-	47.1	-		90.0	95.6	-	
Ever worked as a child day care worker	67.9	1.5 (1.0-2.3)*	1.6 (1.1-2.3)**	28.2	0.7 (0.5-1.0)*		10.0	94.1	0.7 (0.2-2.6)	

^1^Risk behaviour is present if a respondent reported at least one of the behaviours which could increase the risk for toxoplasmosis, listeriosis or CMV infections.

^2^Low level of education: medium- level secondary education or below; medium level of education: higher level secondary education or vocational education; high level of education: diploma level or university education).

OR: Odds Ratio’s; CI: Confidence Interval.

*p-value < 0.10 univariate regression, **p-value <0.05 multivariate regression, ***this variable was excluded from the logistic regression analysis due to small numbers.

Multivariate logistic regression showed that respondents who had a high level of education (OR 1.7; 95% CI: 1.1-2.6; p = 0.01), the Dutch nationality (OR 2.1; 95% CI: 1.0 – 4.3; p = 0.05), did not take folic acid during their first trimester of pregnancy (OR 2.7; 95% CI: 1.4-5.3; p = <0.01), and worked or had ever worked in a children day-care setting (OR 1.6; 95% CI: 1.1-2.3; p = 0.03) were more likely to practice risk behaviour during pregnancy associated with toxoplasmosis (Table 6).

Respondents who were in their third trimester (OR 1.4; 95% CI: 1.1-1.8; p = 0.01) were more likely to practice risk behaviour associated with listeriosis (Table 6).

Respondents who were in their third trimester of pregnancy (OR 0.4; 95% CI: 0.1 – 1.0; p = 0.05) were less likely to practice risk behaviour associated with CMV (Table 6).

## Discussion

Our observational survey regarding knowledge and risk behaviour related to toxoplasmosis, listeriosis and CMV infections during pregnancy showed that - although there was limited knowledge about specific practices to prevent each of these infections - the majority of respondents reported they practiced appropriate behaviour to prevent toxoplasmosis and listeriosis, but not to prevent CMV.

A strength of this study is its large sample size (n = 1,097) and high response rate (66.0%). This study observed both knowledge about preventive practices and the actual risk behaviour of pregnant women regarding preventable infectious diseases, which has not been conducted by many other studies. However, the study population was not representative for all pregnant women in the Netherlands. The study population included mainly higher educated women and women without the Dutch nationality were under-represented [30,31]. This could be partly due to the fact that the questionnaire was not presented in other languages than Dutch. It is also possible that women who did not know the answers did not return the questionnaire, which may have resulted in an overestimation of knowledge levels. In addition, the behavioural questions may have contributed to social desirability bias, which may have affected the frequency of of reported risk behaviour. Another potential weakness of this study is that only respondents who had children aged less than five years answered the questions about preventive behaviours regarding CMV infections. By doing this we could have missed important information from other respondents who are also at risk for CMV infections, like women who work in a children-day care setting or other health care setting. It needs also to be mentioned that we considered risk behaviour to be present if a respondent reported one of the included risk behaviours at least once during their current pregnancy. This may indicate that the many respondents who had undertaken a risk behaviour were actually not frequent risk takers, but had a single exposure. In addition, as this study had a cross-sectional design, the interpretation of associations should remain with caution.

This study indicates that health care professionals play an important role in informing women about preventable infectious diseases as many respondents reported having received information about toxoplasmosis, listeriosis or CMV from their health care provider. Books, magazines and the Internet were also important sources of information for the respondents. Most pregnant women in the Netherlands receive a brochure entitled “Pregnant” during their first antenatal care visit, which is developed by several Dutch organizations involved in mother and child care. This brochure includes information on listeria and toxoplasmosis, but not on CMV infections, which could partly explain the general lack of knowledge of the respondents about CMV. Confirmed by other studies, this study revealed lower median knowledge scores for preventive practices for toxoplasmosis among respondents who had less formal education, had an unplanned pregnancy, were single, had smoked during pregnancy and had never worked as a health care professional. For listeriosis lower level of median knowledge score were seen among respondents who were younger than 25 years, had less formal education, had an unplanned pregnancy, were single, experienced their first pregnancy, had smoked during pregnancy and had never worked as a health care professional [5,7,10]. In addition to these two infectious diseases, this study revealed that respondents with the Dutch nationality and respondents who spoke Dutch at home, had less knowledge about CMV preventive practices. Women with a non-Dutch nationality may have had more knowledge, and thus be more aware, of CMV infections, because the maternal and congenital CMV prevalence is higher among immigrants than among native mothers [22,32]. Counter to our hypothesis and confirmed by another study [5], disease specific knowledge was not necessarily associated with preventive behaviour during pregnancy, regarding toxoplasmosis and listeriosis. And conversely, a lack of knowledge was not always associated with engaging in risk behaviour. Infection with toxoplasmosis during pregnancy is highly associated with eating raw or undercooked meat [12]. And while only half of the respondents demonstrated knowledge of this relationship, the majority indicated that they avoided the behaviour. These results are comparable with alcohol consumption during pregnancy. Many women know they should not drink alcohol during pregnancy, but they do not exactly know the effects of alcohol on the foetus [33]. Contrarily, although there was an overall good understanding that toxoplasmosis could be prevented by gardening with gloves, only one fifth of the respondents did garden without gloves during their pregnancy. For six out of the eight included risk behaviours, there were almost no differences in reported risk behaviours between respondents who were or were not aware of the practices to prevent infectious diseases.

Respondents with a higher educational level, who had the Dutch nationality, who did not take folic acid in their first trimester or ever worked in a children day-care setting had greater odds to report a risk behaviour for toxoplasmosis. Respondents who were in their third phase of pregnancy had higher odds to report a risk behaviour for listeriosis, but lower odds to report a risk behaviour for CMV. These factors indicate that health care professionals involved in mother and child care should give more attention to these women with regard to infectious disease prevention. Behaviour change depends on a range of factors, including the perceptions of the threat [34]. It is possible that women with a higher education are more aware of the low risk of contracting an infectious disease during pregnancy. Pregnant women receive large amounts of information during their first prenatal visit, including methods to prevent infectious diseases and as the amount of information increases, cognitive shortcuts could arise. This may imply that it is more effective for health care professionals to inform pregnant women briefly about behaviours and lifestyle habits they should adopt or avoid and that it may not be necessary to give information on specific infectious diseases. In addition, it could be helpful to repeat some information on preventive practices during a later stage in pregnancy, than only during one of the first prenatal visits. Some preventive methods need more emphasis in prenatal health care, because these were not well adopted by pregnant women in this current study. These preventive methods concern hygienic behaviours in general (e.g. not sharing utensils or cups with children and hand washing after diaper change) to prevent CMV infection, washing or peeling raw fruits and vegetables to prevent toxoplasmosis and properly reheating ready to eat foods to prevent listeriosis.

Concerning CMV, respondents who had less knowledge about the preventive practices did not report more often risk behaviour than respondents with knowledge about the preventive practices. There was a general lack of knowledge, illustrated by the fact that only one eighth of the respondents had ever read, seen or heard anything about CMV and the majority of respondents did not adopt methods to prevent CMV infections. Women within certain professions such as children-day-care workers have a 5 to 25 fold higher risk of acquiring a primo CMV infection during their pregnancy compared to women not in contact with young children [35,36]. However, this study did not find differences in behaviour between respondents who ever worked in a health care or in a children day-care setting and respondents who did not. An explanation for the general lack of knowledge and lack of adopting behaviours towards CMV infection prevention is that health care workers pay little attention to CMV infection [21,27,35]. Another study showed that Dutch doctors involved in mother and child care had suboptimal knowledge on CMV themselves, and they seemed to underestimate the prior risk for a child with congenital CMV infection in their practice [27]. However, it may be important for health care professionals to give information on CMV prevention to pregnant women in the future as a study in France showed that simple information on basic hygiene measures given to women at the beginning of their pregnancy could significantly reduce the incidence of maternal infection during pregnancy [36,37]. In addition, a recent study showed a congenital CMV birth prevalence rate of 0.54% in the Netherlands [22], which is higher than the birth prevalence rate of 0.09% [32] showed in an earlier study and on which many Dutch professional educational materials and guidelines are based [22].

Pregnant women seemed to appropriately avoid risk behaviour without exactly knowing why they avoid it. This could reflect the use of cognitive shortcuts, where complex tasks are reduced to simpler operations which allows people to make rapid, efficient, but sometimes irrational choices [38,39].

Some studies suggest that written education is less effective to establish behavioural change than when health care providers inform clients orally about correct behaviour [40-42]. However, this study did not find any difference in the occurrence of risk behaviour between those we received information on the infectious disease from their health care professional and those who received information through other sources. It would be interesting to investigate what kind of information health care professionals involved in mother and child care give orally and what kind of information they give in written materials about preventable infectious diseases.

## Conclusions

In conclusion, a substantial part of the pregnant women in our study had never heard of listeriosis, toxoplasmosis or CMV, or did not know how to avoid these infections during pregnancy. However, many pregnant women were appropriately avoiding risk behaviours, without knowing what they are avoiding. It remains important that health care providers continue advising pregnant women about behaviours and lifestyle habits which can prevent infectious diseases, but it may be less important to inform pregnant women about specific infectious diseases. In addition, other sources of information about the prevention of infectious diseases in pregnancy must be complete and adequate. In general, more attention towards CMV infection prevention is necessary to improve the knowledge and adoption of the behaviours to prevent CMV infections among pregnant women.

## Competing interests

The authors declare they have no competing interests.

## Authors’ contributions

MTRP, JM, ERS, FGS and EKH developed the protocol. MTRP, JM, ERS and FGS developed the questionnaire about infectious diseases. MTRP collected the data in this study. MTRP, JM and ERS were responsible of data linkage of the two data sources. MTRP, JM, FGS and EKH supported the data analyses. All authors contributed to the editing of the manuscript and have reviewed and approved the final version.

## Pre-publication history

The pre-publication history for this paper can be accessed here:

http://www.biomedcentral.com/1471-2393/13/98/prepub
